# Mitochondrial DNA Release Contributes to Intestinal Ischemia/Reperfusion Injury

**DOI:** 10.3389/fphar.2022.854994

**Published:** 2022-03-16

**Authors:** Shishi Liao, Jie Luo, Tulanisa Kadier, Ke Ding, Rong Chen, Qingtao Meng

**Affiliations:** ^1^ Department of Anesthesiology, Renmin Hospital of Wuhan University, Wuhan, China; ^2^ Department of Anesthesiology, East Hospital, Renmin Hospital of Wuhan University, Wuhan, China

**Keywords:** mitochondrial DNA1, damage-associated molecular patterns2, inflammation3, ischemia/reperfusion injury4, intestinal barrier function5, systemic inflammatory response syndrome6, multiple organ dysfunction syndrome7

## Abstract

Mitochondria release many damage-associated molecular patterns (DAMPs) when cells are damaged or stressed, with mitochondrial DNA (mtDNA) being. MtDNA activates innate immune responses and induces inflammation through the TLR-9, NLRP3 inflammasome, and cGAS-STING signaling pathways. Released inflammatory factors cause damage to intestinal barrier function. Many bacteria and endotoxins migrate to the circulatory system and lymphatic system, leading to systemic inflammatory response syndrome (SIRS) and even damaging the function of multiple organs throughout the body. This process may ultimately lead to multiple organ dysfunction syndrome (MODS). Recent studies have shown that various factors, such as the release of mtDNA and the massive infiltration of inflammatory factors, can cause intestinal ischemia/reperfusion (I/R) injury. This destroys intestinal barrier function, induces an inflammatory storm, leads to SIRS, increases the vulnerability of organs, and develops into MODS. Mitophagy eliminates dysfunctional mitochondria to maintain cellular homeostasis. This review discusses mtDNA release during the pathogenesis of intestinal I/R and summarizes methods for the prevention or treatment of intestinal I/R. We also discuss the effects of inflammation and increased intestinal barrier permeability on drugs.

## Introduction

Mitochondria are the power plants of eukaryotic cells and are involved in the processes of cell proliferation, differentiation, signal transmission, apoptosis, the tricarboxylic acid cycle, and oxidative phosphorylation. Recent studies have shown that when pathogenic microorganisms infect host cells, mtDNA can activate the TLR-9, NLRP3 inflammasome, and cGAS-STING signaling pathways to induce the host innate immune response, which is essential for protection against infections (Chan, 2006). MtDNA contains many unmethylated CPG sequences. Damaged mtDNA release activates pattern recognition receptors (PRRs), which promote inflammation amplification. Intestinal reperfusion injury is the secondary injury occurring after intestinal I/R. It involves intestinal mucosal barrier damage and bacterial transfer. It is accompanied by increases in ROS levels and easily triggers SIRS and MODS (Wu et al., 2013; Zhu et al., 2017; Sun et al., 2018). Its pathogenesis is complex and includes oxygen-free radical damage, calcium overload, and inflammatory reactions. Studies have shown that mtDNA is closely related to intestinal barrier dysfunction. We focus on the close link between mitochondrial quality control and mitochondrial dynamics. Mitophagy eliminates dysfunctional mitochondria, and mitophagy deficiency exacerbates dysfunction.

### Intestinal I/R Injury

Intestinal I/R injury is common in the clinic. Many pathological processes are associated with intestinal I/R injury, such as volvulus, intussusception, incarcerated hernia, acute intestinal ischemia, shock, trauma; certain surgical procedures, such as bowel resection and transplantation, are also associated (Acosta, 2010; Stone and Wilkins, 2015). Intestinal I/R is a common pathophysiological process in many diseases and is closely related to the structural characteristics of the intestine. First, the small intestine is supplied mainly by the superior mesenteric artery, and the nerve endings of the small intestine communicate directly with the arterioles and veins. Second, in various emergencies, the body prioritizes the supply of blood to vital organs such as the heart and brain, and the supply of blood to the gastrointestinal tract is correspondingly reduced, which contributes to the occurrence of intestinal ischemia, increases intestinal permeability, and weakens intestinal barrier function (Liu et al., 2016). Intestinal mucosal barrier dysfunction and increased permeability enable numerous bacteria or toxins to enter the blood or lymphatic channels in the circulatory system, leading to SIRS. When treatment is inappropriate or not timely, the condition eventually develops into MODS (Wu et al., 2017). Extensive intestinal epithelial cell death is a major cause of intestinal mucosal barrier dysfunction, and further development leads to systemic inflammation and distal organ dysfunction (Cheng J. et al., 2013). Microcirculation disturbance and organ injury after intestinal I/R are complicated pathological processes that mainly involve metabolic injury and oxidative stress during ischemia and reperfusion. During ischemia, vascular closure or obstruction leads to a lack of oxygen and nutrients in cells, impairing the expression of mitochondrial respiratory chain ATP synthase (Lin et al., 2013; Tu et al., 2013; Li et al., 2014a; He et al., 2014) and resulting in a decrease in ATP synthesis coupled with continued depletion of ATP, leading to ATP deficiency (Kalogeris et al., 2012). Microcirculatory dysfunction induced by I/R injury may lead to multiple organ damage (Eltzschig and Eckle, 2011), organ fibrosis (Rai et al., 2017), and even organ failure (Katseni et al., 2015), whose prevention and treatment are critically important.

## Possible Mechanisms by Which MODS Is Induced by Intestinal I/R Injury

**FIGURE 1:**
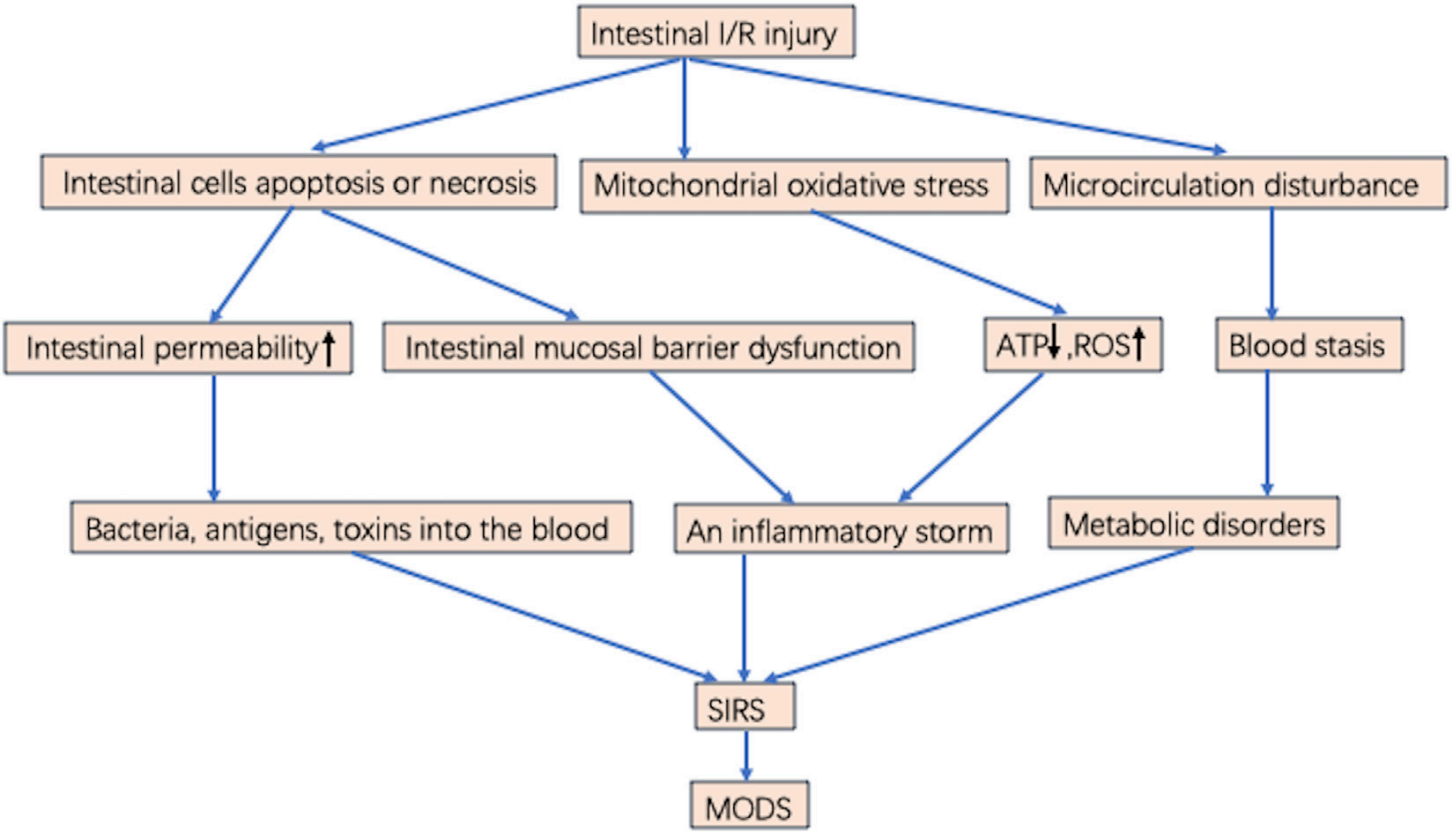
Possible mechanisms of MODS induced by intestinal I/R injury. Intestinal I/R injury results in intestinal cells apoptosis or necrosis, mitochondrial oxidative stress, and microcirculation disturbance. microcirculation disturbance induces blood stasis and metabolic disorders. Increasing ROS, cytokines, chemokines, and inflammatory mediators lead to an inflammatory storm. Intestinal mucosal barrier dysfunction and increased permeability result in numerous bacteria or toxins through the blood into the circulatory system, leading to SIRS. When the treatment is inappropriate or not timely, it eventually develops into MODS.

Superior mesenteric artery ischemia or occlusion produces gradient ischemia along the long axis of the intestinal tract. Figure 1 Ischemia is most severe at the distal end of the small intestine and near the end of the colon, while blood flow in the middle and far end of the colon is largely unaffected, with ischemia located in the mucosa and submucosa rather than in the muscularis and serosa (Cerqueira et al., 2005; Ramos et al., 2016). Intestinal I/R injury destroys the tight connections of mucosal epithelial cells. Bacteria and enterotoxins enter the lymphatic circulation and blood circulation through the damaged mucosal barrier. Sepsis and multiple organ failure may occur when intestinal tissue ischemia is severe or the area of ischemia is extensive (Souza et al., 2004; Kinross et al., 2009). Local I/R injury can lead to distant organ damage and multiple organ failure. Intestinal I/R injury leads to remote organ injury (Santora et al., 2010). Lung function is susceptible to damage from distant organ ischemia; thus, remote organ injury is prone to develop into acute respiratory distress syndrome (ARDS) and SIRS, eventually progressing to MODS. If the body is in an inflammatory state, local and distant cellular responses are amplified, exacerbating primary ischemia, which is the stage of postreperfusion intestinal injury (Courties et al., 2014). In addition, after the gut barrier is damaged, bacteria initiate and maintain the production of local inflammatory mediators. Bacteria and endotoxins enter the intestinal stroma, lymphatic vessels, and blood circulation from the intestinal lumen and travel to distant organs, which plays an important role in the development of MODS (Stallion et al., 2005; Deitch, 2012) Figure 1.

The extent of tissue or cell injury following intestinal I/R and the progression to irreversible injury are directly related to ischemia duration (Abela and Homer-Vanniasinkham, 2003). This suggests that restoring blood flow as soon as possible may limit cell damage and death. Almost all organs have a common response to reperfusion injury including processes such as activation of endothelial cells, mononuclear macrophages, hypertrophic cells, and neutrophils and release of reactive oxygen species (ROS), cytokines, chemokines, and many inflammatory mediators, which mediate and activate multiple signaling pathways forming a cellular network and biomediator network. This response causes an inflammatory storm leading to SIRS and progressing to sepsis or MODS. This extends hospital stays and increases in-hospital mortality in critically ill patients. Intestinal I/R injury can cause MODS by disrupting the balance between inflammatory and anti-inflammatory responses, breaking down the intestinal barrier, increasing intestinal permeability, and promoting metabolic disorders and oxidative stress in ischemic cells.

### Systemic Inflammatory Response Syndrome

Inflammation plays an important role in I/R. Neutrophils and white blood cells are recruited to produce cytokines, chemokines, and inflammatory mediators, which induce inflammatory responses (Kvietys and Granger, 2012; Frangogiannis, 2015). During reperfusion, the immune cells delivered by the blood reenter the tissue, and leukocyte sequestration increases significantly. To save ischemic tissue, cell metabolism should be accelerated, but during reperfusion, the influx of oxygen into the blood promotes the production of ROS by xanthine oxidase (XO) and NADPH oxidase. Necrotic cells in ischemic tissue emit danger signals, and adhesion mediators are formed between innate immune cells and postcapillary veins. Finally, neutrophils are isolated into ischemic tissue. Neutrophils activate NADPH oxidase-dependent respiratory bursts, release hydrolases, produce highly toxic hypochlorite and N-chloramine through MPO enzyme activity, and secrete pore-forming molecules, causing extensive collateral damage to blood vessels and parenchymal cells. Infiltrating neutrophils induce reperfusion damage that amplifies ischemia-induced cell damage (Ford, 2010; Kvietys and Granger, 2012; Frangogiannis, 2015).

Studies have shown that SIRS can directly cause MODS. SIRS refers to stimulation, by severe infectious or noninfectious factors, of the activation of inflammatory cells. This stimulation producing many inflammatory mediators, to which the body has a systemic inflammatory cascade response. MODS, caused by an uncontrolled inflammatory response, is related not only to the overexpression and secretion of inflammatory mediators but also to the abnormal production of host anti-inflammatory mediators of endogenous inhibitors. The body produces endogenous anti-inflammatory responses that decrease immune function and increase susceptibility when the body is injured or infected. In 1996, Bone RC introduced the concept of compensatory anti-inflammatory response syndrome (CARS) (Bone, 1996). Physiologically, proinflammatory and anti-inflammatory processes exist in a balanced state; when proinflammatory processes become dominant, the external response and cell damage are induced, which manifest as SIRS. In contrast, when anti-inflammatory processes predominate, the host-to-external stimulus response is low, which increases susceptibility to infection and performance similar to CARS. When both are hyperactive, the immune system exhibits are more serious disorder known as mixed anti-inflammatory response syndrome (MARS). Either CARS or MARS reflects disorder of the body’s inflammatory response and destruction of the internal environment, laying the foundation for MODS. Infection-related or non-infection-related factors can activate inflammatory cells to release a variety of cytokines and inflammatory mediators, such as IL-1, IL-6, IL-8, IL-12, TNF-α, NO, leukotriene B4, IFNα, and IFNβ (McNab et al., 2015; Chen et al., 2017) to participate in the body’s inflammatory immune response in order to resist external pathogenic factors. Anti-inflammatory cytokines such as IL-4, IL-5, and IL-13 also increase significantly to prevent excessive inflammatory injury (Wojdasiewicz et al., 2014; Kalogeris et al., 2016; Boshtam et al., 2017). When inflammatory and anti-inflammatory responses are out of balance, multiple inflammatory mediators form a cascade effect. The interactions of these mediators induce a variety of pathophysiological processes, including the endothelial cell inflammatory response, increased vascular permeability, inflammatory infiltration, and tissue damage. This eventually leads to the occurrence of MODS.

### Intestinal Barrier Impairment and Increased Permeability

In the physiological state, the intestinal mucosal barrier acts as a filter for the gut, enabling the absorption of nutrients and preventing bacteria, macromolecules, and toxic compounds from entering the internal environment (Camilleri et al., 2012). Intestinal epithelial cells, microbes, and the immune system work together to maintain intestinal homeostasis. Impairment of intestinal integrity is an important cause of SIRS and MODS. Breakdown of the intestinal barrier can lead to local intestinal dysfunction and absorption of toxic substances into the blood, resulting in bacterial translocation and systemic abnormalities such as sepsis (Assimakopoulos et al., 2018). These changes lead to increased intestinal permeability. Bacteria, antigens, and toxic substances activate mucosal membrane immune responses through the intestinal mucosa, leading to abdominal pain and diarrhea (Farhadi et al., 2007). These inflammatory mediators signal to epithelial cells, nerve cells, and muscle cells, leading to intestinal dysfunction (Macdonald and Monteleone, 2005). Increased intestinal permeability under conditions of severe trauma, shock, and critical conditions and barrier damage maintain an inflammatory environment, leading to changes in microbial toxicity and acute gastrointestinal injury (Piton et al., 2011). As intestinal permeability increases, proinflammatory cytokines such as TNF-α, IL-1, and IL-6 are released into the systemic circulation, fluid seeps out of the intestine, and angioedema occurs (Angus and van der Poll, 2013). Proinflammatory cytokines also induce changes in intestinal tight connection proteins, further increasing intestinal permeability (Shea-Donohue et al., 2010; Zhou et al., 2015). In addition, the toxicity and invasiveness of the gut microbiome increases, and translocating bacteria, cytokines, and endotoxins are released from the intestine. These factors cause damage to distant organs, so the intestine is considered to be the origin of sepsis and MODS (Deitch, 2010).

### Disturbance of Microcirculation and Metabolism

A series of pathophysiological changes and abnormal cell metabolism occur in intestinal tissue due to ischemia and hypoxia. On the one hand, microcirculation disorder manifests as microcirculation congestion, and blood flow stasis causes tissue hypoxia to produce metabolic acidosis, induces intravascular coagulation, and forms microthrombi. Microthrombi aggravate hypoxia and metabolic acidosis of tissues and organs, forming a vicious cycle. Severe hypoxia and acidosis can damage vascular endothelial cells, increase vascular permeability, and cause widespread edema in tissue. In addition, the stability of lysosomes is destroyed, the lysosomal membrane ruptures, and cells undergo self-soluble necrosis. These processes work together to cause systemic multiple organ dysfunction. On the other hand, histiocyte metabolism is affected. Metabolic disorders occur due to hypoperfusion and hypoxia of the gut. The body is in a state of stress; therefore, catecholamine and adrenal corticosteroid release increases, metabolic decomposition is exacerbated, and energy consumption significantly increases. Under hypoxia, cell anaerobic metabolism increases, and lactic acid accumulates, leading to metabolic acidosis.

### Mitochondrial Oxidative Stress and Intestinal I/R

The human intestine is rich in mitochondria, which participate in numerous redox reactions and produce ATP by oxidative phosphorylation. Such energy metabolism is the main source of oxidants. Studies have shown that mitochondria are major participants in the process of intestinal oxidative stress. When the intestine is exposed to oxidative stress, it produces many reactive oxygen species (ROS) and reactive nitrogen species (RNS). This causes the body’s oxidant and antioxidant defenses to be out of balance, leading to aging and disease. ROS and RNS come from many different sources, including mitochondrial respiratory electron transport chains, xanthine oxidase, NADPH oxidase, and the NO synthetase system (Hu C. et al., 2019). In intestine cells, mitochondria are the main sources of ROS, mainly mitochondrial respiratory chain enzyme complex I and complex III (Rabbani and Thornalley, 2019). When the process of mitochondrial oxidative phosphorylation is disturbed, the formation of ATP is reduced significantly, and the propensity for ROS formation is increased (Toldo et al., 2018). In the physiological state, the body has many ways to regulate the oxidative stress produced by mitochondrial ROS, such as the superoxide dismutase system, which can convert reactive superoxide radicals into hydrogen peroxide and detoxify it further by enzyme catalysis. In the case of intestinal ischemia, the restoration of blood supply increases ROS, leading to mitochondrial dysfunction and intestinal mucosal cell damage. The inflammatory response eventually disrupts intestinal mucosal barrier function.

In addition, mitochondria can not only produce energy but also regulate calcium homeostasis. Na^+^-K^+^-ATPase and Ca^2+^-ATPase, which are essential for maintaining the normal structure and function of mitochondria, are located on the mitochondrial membrane. When the activity of the two ion pumps changes, mitochondrial membrane permeability transition pores can be activated, resulting in the functional and structural breakdown of mitochondria and causing cell death (Yue et al., 2012). Other studies have confirmed that when hypoxia occurs in the intestine, the activity of the mitochondrial respiratory chain enzyme complex decreases. This produces large numbers of ROS and significantly reduces ATP synthesis (Duchen, 2004). In addition, the atpase activity in intestinal mitochondria changes, Na^+^-K^+^-ATPase activity decreases, and Ca^2+^-ATPase activity increases (Baines, 2009). Changes in mitochondrial membrane potential cause dysfunction, lead to the release of large amounts of ROS and apoptosis-promoting factors, and eventually induce apoptosis or necrosis of intestinal cells (Poyton et al., 2009). When intestinal I/R occurs, the amount of mtDNA in the circulation also increases significantly. Hu et al. (Hu et al., 2018a) revealed the leading role of mtDNA in the pathogenesis of intestinal I/R injury. A few experimental results have shown that after reperfusion of the ischemic intestine, mitochondrial respiratory function and transmembrane potential are decreased, mitochondrial membrane permeability is increased, and stability is decreased.

During the ischemia or hypoxia period, electron flow through the mitochondrial respiratory chain is inhibited, and ATP synthetase cannot phosphorylate ADP to produce ATP (Grover et al., 2004; Di Lisa et al., 2007). Ischemia or hypoxia also affects ATP hydrolase, which hydrolyzes the remaining ATP (Murphy and Steenbergen, 2008). As a result, ATP levels drop rapidly during ischemia. Mitochondria are important sources of oxidative stress in intestinal I/R. Excessive ROS are produced by the electron transport chain, mitochondrial outer membrane proteins, and other mitochondrial proteins. Under physiological conditions, superoxide produced by electron transport chain complexes I and III is neutralized by SOD. However, in ischemia, mitochondrial complex I superoxide leakage increases, breaking the dynamic balance between cell oxidation and antioxidants and thus aggravating cell damage (Lee et al., 2012; Dan Dunn et al., 2015). During ischemia, the mitochondrial permeability transition pore (mPTP) is inhibited by acidosis and is at rest. I/R induces calcium overload in mitochondria, and the production of ROS causes the mPTP to open (Di Lisa et al., 2009; Ong and Gustafsson, 2012; Di Lisa et al., 2017). Thus, molecules with small molecular weights can pass through this pore, and many H^+^ ions enter the mitochondrial matrix, thereby dissipating the mitochondrial membrane potential, uncoupling the electron transport chain, and inhibiting ATP synthesis (Baines, 2010). At the same time, water seeps into the organelles, causing them to swell and rupture.

When the blood supply to the ischemic organs is restored, a large amount of oxygen influx increases the overproduction of ROS. In recent years, it has been increasingly recognized that RNS also contribute to I/R injury (Duan and Kasper, 2011; Kvietys and Granger, 2012; Raedschelders et al., 2012). ROS refer to free radicals and non-free radicals with relatively strong oxidation and reduction properties, such as superoxide anions (O_2_^−^), hydrogen peroxide (H_2_O_2_), and hydroxy free radicals (OH^−^). RNS is a series of complexes produced by NO metabolism, with NO as the center, including peroxynitrite anion (ONOO^−^), nitrate anion (NO^−^), nitrogen dioxide (NO_2_), and others. The effects of ROS and RNS are related to their concentrations *in vivo*. At moderate levels, they act as mediators in signal transduction. However, when their concentrations increase markedly, these species are formidably destructive to lipids, proteins, nucleic acids, and the extracellular matrix. Reactive nitrogen and oxygen species (RNOs) promote reperfusion injury by altering the structure or function of macromolecules, disrupting or activating signal cascades, stimulating the production and release of proinflammatory mediators in various cell types, and inducing the expression of adhesion molecules.

## mtDNA Release and Intestinal I/R

Mitochondria are divided into the mitochondrial outer membrane, mitochondrial interspace, mitochondrial inner membrane, and mitochondrial matrix. Mitochondrial DNA is a small double-stranded circular molecule with a low degree of methylation. It is a regulator of cell death, inflammation, and oxidative stress and can promote oxidative stress, inflammation, and apoptosis if mitochondrial dysregulation or dysfunction occurs. Mitochondrial DNA released after intestinal I/R injury has been found to cause an inflammatory response and intestinal barrier dysfunction (Wu et al., 2017). MtDNA is in the mitochondrial matrix. After mitochondrial damage, mtDNA is released into the cytoplasm through a variety of mechanisms. How does mtDNA reach the cytoplasm from the mitochondrial matrix? The first step is activation of the mPTP. Activation of the mPTP has been shown to help release mtDNA during cell death and mitochondrial injury (García and Chávez, 2007). The opening of the mPTP in the first several minutes of reperfusion is an important determinant of I/R injury (Wang et al., 2016). The release of mitochondrial components is associated with mPTP activation. Second, mitochondrial-derived vesicles (MDVs) mediate the transfer of mitochondrial proteins to endosomes, thereby facilitating antigen presentation, which can introduce mitochondrial DNA into the endocytosis mechanism (West and Shadel, 2017). Therefore, mitochondrial DNA can be released directly into the cytoplasm via MDVs. In addition, the regulatory proteins BAX and BAK monitor the release of mtDNA. White et al. (White et al., 2014) found that BAX and BAK induce mitochondrial membrane permeability and that mtDNA is then exposed. Oxidative stress and cell death are key to the release of mtDNA (Hu et al., 2019c).

Studies have found that mitochondrial DNA is released after mitochondrial outer membrane permeabilization (MOMP). How does this process work? In the process of apoptosis, the apoptotic genes BAX and BAK form oligomers on the outer membrane, leading to MOMP. The mitochondrial interstitial protein is then released, which activates caspase proteases to initiate apoptosis. This is a noninflammatory form of cell death that does not trigger an immune response (Arandjelovic and Ravichandran, 2015). However, when caspase activity is blocked after MOMP occurs, the cells activate inflammation and the type I interferon response through the mtDNA signaling-cGAS-STING pathway, leading to cell death (Giampazolias et al., 2017). How mtDNA reaches the cytoplasm still needs to be addressed. After the occurrence of MOMP, the mitochondrial outer membrane forms pores. Interestingly, the sizes of BAX-mediated pores change dynamically. After the occurrence of MOMP, the mitochondrial outer membrane pores widen over time, resulting in mitochondrial membrane extrusion. Experiments by Riley JS et al. have demonstrated this process (Riley et al., 2018). Its existence means that after MOMP, the mitochondrial inner membrane can be extruded through the BAX-mediated outer membrane holes. When the mitochondrial membrane enters the cytoplasm, mitochondrial inner membrane permeabilization (MIMP) occurs, and mtDNA is released into the cytoplasm.

## MtDNA and Signaling Pathways

**FIGURE 2:**
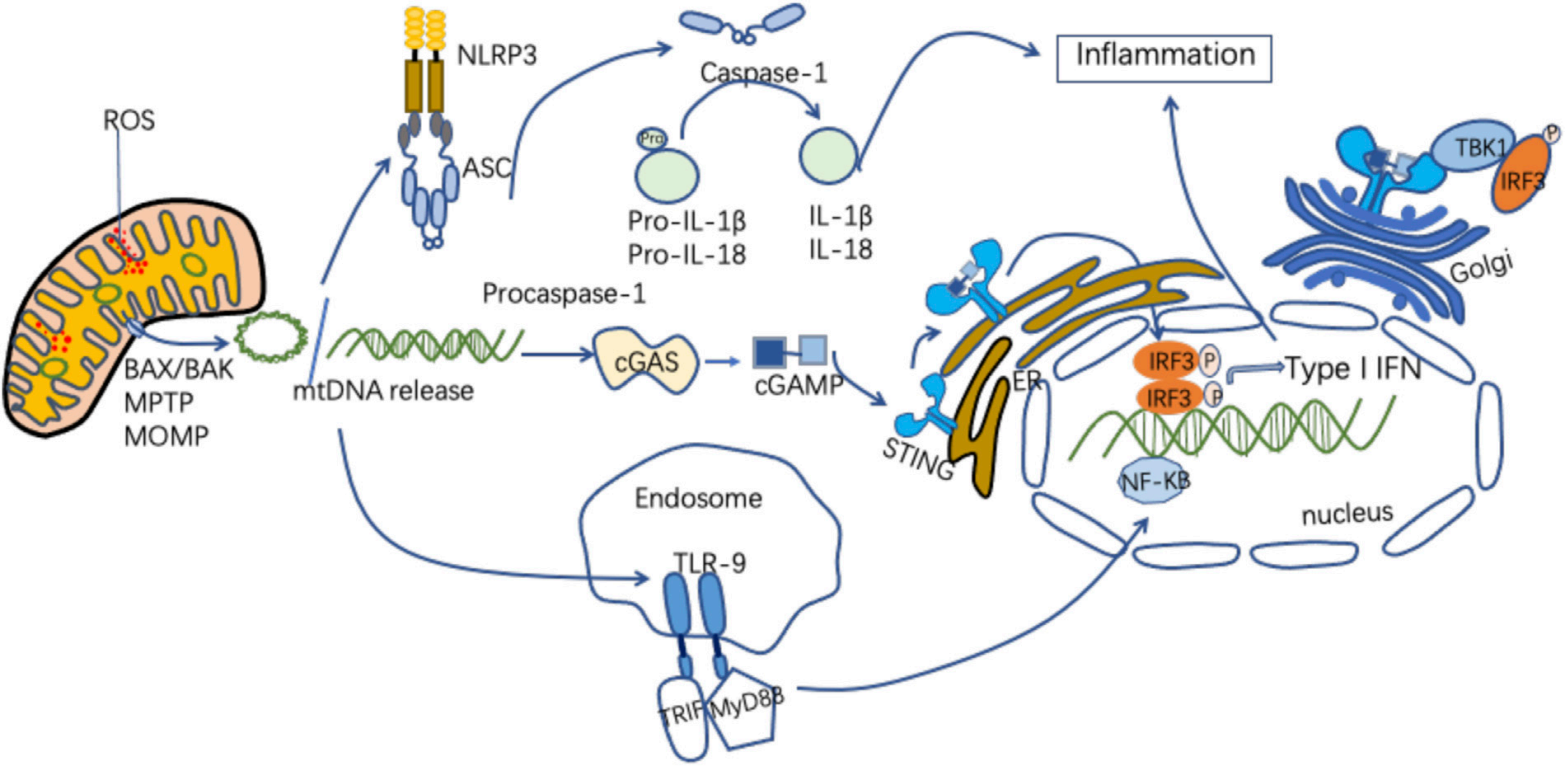
Mechanisms of released mtDNA activation of inflammation. MtDNA can be released as circular molecules or DNA fragments when mitochondria are damaged or stressed. MtDNA release can be mediated through the mPTP, the MOMP, and the apoptosis-associated protein BAX/BAK. The released mtDNA is involved in the inflammatory response through a variety of signaling pathways. MtDNA binds to NLRP3, activating the NLRP3 inflammasomes, which induces caspase-1 activation, causing the maturation and secretion of pro-inflammatory cytokines and participating in the inflammatory response. MtDNA binds to cGAS to form cGMP-AMP (cGAMP), which activates STING, and the activated STING recruits and activates TBK1. This leads to IRF3, NF-KB phosphorylation and nuclear translocation, promoting increased expression of interferon and inflammatory genes. MtDNA binds to TLR9, promoting MyD88 pathway activation and participating in inflammatory cytokine transcription.

As an important DAMP, mtDNA can activate innate immune and inflammatory responses in a variety of ways (Figure 2). Figure 2 MtDNA mediates inflammation and participates in the pathogenesis of disease through multiple signaling pathways, including intestinal I/R injury pathways (Zhang et al., 2019). MtDNA in the gut exacerbates inflammation and gut barrier dysfunction during intestinal I/R injury (Hu et al., 2018a).

### MtDNA and TLR-9

TLR-9 is a protein receptor encoded by the TLR-9 gene, which is mainly expressed in immune cells such as monocytes, macrophages, and dendritic cells. It is highly conserved and mainly recognizes the DNA of bacteria and viruses (Hemmi et al., 2000). MtDNA is composed of unmethylated CpG dinucleotides and is released when mitochondria are damaged. TLR-9 is located in the endoplasmic reticulum and can be activated by CpG DNA (Barbalat et al., 2011). MtDNA is a major type of DAMP that recognizes TLR-9 of immune cells and activates the body’s innate immune response (Kausar et al., 2020). After the released mtDNA recognizes TLR-9, it induces a strong inflammatory response in the body (Wei et al., 2015). MtDNA binds to TLR-9, recruits myeloid differentiation factor 88 (MyD88), activates MAPK and NF-kB, and induces secretion of proinflammatory cytokines (Riley and Tait, 2020). Zhang et al. observed that mtDNA is released into the circulation during SIRS and activates TLR-9 on neutrophils (Zhang et al., 2010a; Zhang et al., 2010b). Various studies have shown that there is a strong correlation between the levels of mtDNA and TLR-9 and that increasing the degradation of mtDNA can reduce the inflammatory response that depends on TLR-9 (Oka et al., 2012). A few studies have shown that intravenous injection of damaged mitochondrial fragments containing a large quantity of mtDNA can stimulate the systemic inflammatory response. Knocking out the MyD88 gene or TLR-9 gene in rats or using TLR-9 antagonists significantly reduces this phenomenon. The significance of TLR9-MyD88 signaling also provides a theoretical basis for inhibiting the systemic inflammatory response. In addition, mtDNA can promote the inflammatory response after I/R injury via TLR9. When cells are hypoxic, mtDNA is complexed with high-mobility group protein B1 (HMGB1) and can autonomously bind to TLR9 to enhance inflammation (Liu et al., 2015). Oxidative mtDNA has been shown to trigger the TLR9 pathway, and oxidative mtDNA released by neutrophils induces plasmacytoid DCs (pDCs) to produce type I interferon (Caielli et al., 2016). Mark et al. reported that the release of mtDNA and TFAM after cell injury, which leads to TLR9-dependent interferon secretion, plays a key role in regulating the sterile immune response (Julian et al., 2013).

### MtDNA and NLRP3

The NLRP3 inflammasome is a kind of N-like receptor (NOD-like receptor, NLR) that is composed of NLRP3, an adaptor protein (ASC), and aspartic protease-1 (Caspase-1). It is a kind of pattern recognition receptor (PRR) in the cell membrane. PRRs can recognize related pathogenic microorganisms. Abnormally activated NLRP3 recruits and activates caspase-1, resulting in the release of IL-1 and IL-18 into the extracellular environment and leading to progression of I/R damage (Guo et al., 2016). In 2011, it was first reported that mtDNA facilitates the activation of NLRP3 inflammation, and many reports have since reinforced the idea that mitochondrial damage and mtDNA release are amplified by activation of NLRP3 inflammation. However, a recent study by Shimada et al. has suggested that NLRP3 may not enhance mitochondrial DNA release but rather stabilize mtDNA in the cytoplasm (Yu et al., 2014; Bronner et al., 2015). Studies have confirmed that NLRP3 is widely present in the epithelial cells of the intestinal tissue, and the NLRP3 inflammasome is activated to participate in inflammatory injury of the intestine. Generally, Caspase-1 exists in an inactive form in the body, and the NLRP3 inflammasome mediates its activation (Tsutsui et al., 2010). MtDNA can directly activate the NLRP3 inflammasome (Huo et al., 2013). Abnormal recruitment of NLRP3 activates Caspase-1, and cells produce mature IL-1β and IL-18, triggering a specific cell death mode called pyroptosis (Guo et al., 2015). In addition, NF-kB can enhance the expression of NLRP3 and promote the upregulation of NLRP3 receptor protein expression. NF-kB plays a key role in regulating proinflammatory factors (Taniguchi and Karin, 2018). TLRs can activate the NF-kB pathway, enhance the expression of NLRP3, and participate in intestinal inflammatory damage. Yang et al. found that after rat intestinal I/R, the expression of p-NF-kB p65, NLRP3, Caspase-1, and inflammatory factors is increased (Yang et al., 2020). This suggests that intestinal I/R may be related to the NF-kB/NLRP3 pathway.

### MtDNA and the cGAS-STING Signaling Pathway

The cGAS-STING pathway regulates the production of type I interferons. STING can be activated by its DNA, which induces the production of type I interferon in a STING-dependent manner (Schoggins et al., 2014). The cGAS enzyme is a type of cytoplasmic DNA that activates STING to induce an innate immune response by producing cGAMP. STING then binds and activates TANK-binding kinase 1 (TBK1). TBK1 phosphorylates IRF3, facilitates its dimerization, and shifts to the cell nucleus, resulting in a type I interferon response (Storek et al., 2015; Chen Q. et al., 2016). Cyclic GMP-AMP synthase (cGAS) is a DNA pattern recognition receptor that can recognize DNA to trigger host innate immunity. MtDNA released by damaged cells is an effective ligand that transmits signals through cGAS (Fang et al., 2016). After cGAS recognizes DNA, it catalyzes the synthesis of cyclic dinucleotides (cyclic GMP-AMP, cGAMP). CGAMP combines with the adaptor protein STING (stimulator of interferon) to activate the cGAS-STING signaling pathway and promote the expression of type I interferon, thereby regulating innate immunity (Sun et al., 2013). The STING pathway activates TBK1 to induce phosphorylation of both the IRF3 and NF-kB pathways, and the expression of IFNs (IFNα and IFNβ) and TNF-α increases (Ishikawa and Barber, 2008). Both IFNs and TNF-α can trigger necroptosis of intestinal epithelial cells (Wallach et al., 2016). Necroptosis can promote intestinal barrier dysfunction after intestinal I/R, and infection can significantly aggravate intestinal I/R injury (Victoni et al., 2010; Mittal and Coopersmith, 2014). STING plays an important role in regulating the homeostasis and integrity of the intestinal barrier (Fischer et al., 2017; Canesso et al., 2018). In the gastrointestinal mucosal system, mtDNA is released when mitochondrial damage is caused by ischemia or hypoxia. It then activates the STING signaling pathway to stimulate the production of inflammatory factors. STING is an important molecule for cytoplasmic DNA to activate the innate immune response. Both endogenous and exogenous DNA are recognized by STING and mediate its downstream inflammatory signals. The cGAS-STING signaling pathway can be regulated by adjusting the phosphorylation and ubiquitination of STING. Mitochondrial oxidative stress induces mitochondrial DNA release after intestinal I/R and subsequently promotes intestinal I/R injury (Hu et al., 2018a). Intestinal I/R injury is an important pathway for acute intestinal barrier damage. The breakdown of the intestinal barrier mediated by STING results in severe sepsis (Hu et al., 2019b). MtDNA induces intestinal necroptosis, further promoting intestinal I/R damage. In addition, mtDNA-mediated STING signaling triggers necroptosis via synergistic IFN and TNF-α signal transduction (Zhang et al., 2020).

Through the above signaling pathways, mtDNA induces inflammatory activation, initiates SIRS, and damages the intestinal mucosal system through the circulatory system (Hu et al., 2018a).

## Mitochondrial Dynamics and Mitophagy

The prevailing view is that mitochondrial dynamics and mitochondrial quality control are closely related. Mitochondrial dynamics refers to the fact that mitochondria are constantly undergoing fission and fusion. Mitochondrial fission facilitates mitochondrial transport, adapts to metabolic demands, and maintains cellular distribution (Michalska et al., 2018; Pagliuso et al., 2018). Mitochondrial fusion is a transient response that enables the exchange of mitochondrial contents (Chen et al., 2010; Tam et al., 2015) and buffers acute mitochondrial damage (Youle and van der Bliek, 2012). When damage exceeds the limits of fusion repair, the damaged mitochondria are cleared via mitophagy (Xian and Liou, 2021). Mitochondrial division/fusion dysregulation alters cellular responses and causes defective mitochondrial signaling, which may lead to mitochondrial-associated diseases (Ban et al., 2010; Suomalainen and Battersby, 2018). Mitochondrial quality control is a protective mechanism, and mitophagy is an important aspect. Mitophagy copes with stress-induced mtDNA damage (Kumar and Jurkunas, 2021).

Whether mitochondrial fission precedes mitophagy has been debated. Many studies have suggested that mitochondrial fission initiates mitophagy (Twig and Shirihai, 2011; Youle and van der Bliek, 2012; Kageyama et al., 2014; Ikeda et al., 2015). The Shirihai group describes the asymmetric fission of mitochondria (Twig et al., 2008). Gottlieb et al. found that mitochondrial membrane potentials differ after fission; mitochondria with high membrane potential fuse with other mitochondria, while those with low membrane potential becoming targets for mitophagy (Gottlieb et al., 2021). Isolated daughter mitochondria produced by mitochondrial fission are cleared by mitophagy (Chen M. et al., 2016; Burman et al., 2017; Kleele et al., 2021). It has recently been proposed that mitochondrial fission is not essential for mitophagy (Xian et al., 2019; Xian and Liou, 2019). Xian et al. found that Drp1-regulated mitochondrial fission has little effect on STX17-induced mitophagy. Fused mitochondria that are deprived of Drp1 can undergo mitophagy (Xian et al., 2019). This finding challenges the idea that mitochondrial fission is a prerequisite for mitophagy (Friedman and Nunnari, 2014). Mitochondrial fusion is a form of self-repair when acute stress occurs in mitochondria. Mitochondrial fission may be inhibited under acute injury when stress accumulates beyond the fusion repair threshold, at which point the fused mitochondria can undergo mitophagy (Xian and Liou, 2021).

Mitophagy is a form of macrophagy, or selective autophagy, of damaged mitochondria (Mohsin et al., 2021). It is usually divided into ubiquitin-dependent and nondependent pathways. In mammals, there are four major mitophagy regulatory pathways in damaged mitochondria. The PTEN-induced PINK1/Parkin pathway regulates ubiquitin-dependent mitophagy. The PINK1/Parkin-nondependent pathway includes receptor-mediated, lipid-mediated, and ubiquitin-mediated mitophagy (Sekine and Youle, 2018; Villa et al., 2018). Mitophagy clears dysfunctional mtDNA and reduces the occurrence of disease caused by mitochondrial damage (Youle and Narendra, 2011). Bit-by-bit mode mitophagy does not remove mtDNA, which is a way to protect undamaged mtDNA (Jian et al., 2018). Disruption of mitophagy leads to dysfunctional mitochondrial accumulation and elevated ROS levels, and treatment with antioxidants alleviates the functional defects in cells (García-Prat et al., 2016). This suggests that decreased mitophagy may exacerbate tissue damage caused by oxidative stress. Mitochondrial damage causes a decrease in the mitochondrial membrane potential, which maintains mitochondrial homeostasis through mitophagy and biogenesis. Defects in the mitophagy process lead to the accumulation of ROS in dysfunctional mitochondria, and mtDNA is released into the cytoplasm, activating inflammatory responses (Nakahira et al., 2011; Deutschman and Tracey, 2014).

## Prevention and Treatment of Intestinal Ischemia/Reperfusion Injury

I/R injury is the result of multifactorial interactions, and dysfunction of cells due to abnormal signaling pathways is one of the major mechanisms of MODS (Ibáñez et al., 2015; Lu et al., 2016; Rossello and Yellon, 2018). Table 1 Thus, a delicate balance between apoptotic and antiapoptotic mechanisms is required (Eltzschig and Eckle, 2011). Combining several protective measures may be an improved strategy for minimizing injury (Table 1).

**TABLE 1:** Ways of prevention and treatment of intestinal ischemia/reperfusion injury.

Therapy method	Mechanism	Step
Ischemic conditioning	Increasing the tolerance of organs and tissues to ischemia and reperfusion	Ischemic preconditioning (IPC)
		Ischemic postconditioning (IPO)
		Remote ischemic preconditioning (RIPC)
Energy therapy	Providing enough ATP to ameliorate the internal environment disorder	Creatine supplements, fructose diphosphate (FDP), ATP
Anti-free radical therapy	Using antioxidants to reduce oxidative damage	ATP, glutathione, melatonin, vitamin C, vitamin E
Anti-leukocyte adhesion therapy	Inhibiting leukocyte activation and adhesion	PAF, anti-CD11 monoclonal antibody
Glucocorticoids	Regulate immunity and suppress inflammation	prednisolone, betamethasone, dexamethasone

### Ischemic Conditioning

The intestinal mucosal barrier is mainly composed of a mechanical barrier, an immune barrier, and a biological barrier. The various parts interact to protect the body. MtDNA release, inflammatory factors, bacterial toxin displacement, and other factors can cause intestinal I/R injury. This in turn destroys the intestinal barrier. Therefore, preventing intestinal I/R injury can greatly reduce the functional damage of the intestinal mucosal barrier. Increasing the tolerance of organs or tissues to ischemia and reperfusion may be an important method of preventing reperfusion injury, especially during major surgeries directly related to ischemia and reperfusion (Hausenloy and Yellon, 2016). Current measures to prevent intestinal I/R mainly include ischemic preconditioning (IPC) and ischemic postconditioning (IPO) (Camara-Lemarroy, 2014; Heusch, 2015). A meta-analysis has revealed that ischemic preconditioning significantly improves tissue tolerance to ischemia (Salvador et al., 2016). Postischemic treatment can significantly reduce the degree of intestinal I/R injury (Jonker et al., 2016). IPC is a clinical application of cell protection for key organs (Li et al., 2013; Li et al., 2014b).

IPC involves short-term ischemia and reperfusion of the intestinal tissue before the expected ischemia period, which can initiate the endogenous protective mechanism to deal with the subsequent ischemia for a prolonged period and reduce the damage to tissues and organs. This is adaptive protective mechanism for a transient ischemic period (Miranda et al., 2019). Many studies have shown that IPC can reduce intestinal tissue damage and alleviate SIRS. Hotter et al. (Hotter et al., 1996) elucidated the protective effect of IPC against intestinal I/R. IPC can reduce intestinal I/R by reducing oxidative stress and participating in signal transduction (Avgerinos et al., 2010; Mallick et al., 2010; Xing et al., 2014). IPC attenuates neutrophil-endothelial cell adhesion cascade reactions; reduces intestinal oxidative damage (Ferencz et al., 2004); and protects the intestine from I/R damage by releasing mast cell degranulation-mediated carboxypeptidase A (Xing et al., 2014), inhibiting heme oxygenase (Mallick et al., 2010), and regulating arachidonic acid cascade reactions (Avgerinos et al., 2010). IPC reduces the release of proinflammatory cytokines (Wang et al., 2015). Results obtained by Ji, Y.Y, et al. (Ji et al., 2015) have shown that IPC can reduce the levels of MPO and TNF α, inhibit the expression of ICAM-1 and VCAM-1, and decrease the activity and expression of NF-kB to protect the intestine from I/R injury. Although the protective effect of IPC on the intestinal tract has been well established, IPC has not been widely used in the clinic. More clinical trials are needed to further study the use of IPC in the intestine.

IPO refers to the use of a few short reperfusion and re-ischemia cycles after intestinal tissue ischemia and before recovery of perfusion, which can increase tissue resistance to I/R injury and protect intestinal tissue. It is a safe and feasible method in the clinic. Studies have revealed that ischemic posttreatment can effectively improve the morphology and respiratory function of intestinal mucosal cell mitochondria and increase mitochondrial transmembrane potential, suggesting that the protective effect of ischemic posttreatment may be related to mitochondria. Cheng, C.H, et al. (Cheng CH. et al., 2013) demonstrated the protective effect of IPO against intestinal I/R injury in rats. IPO was found to alleviate intestinal mucosal injury and oxidative stress by regulating mPTP formation to ameliorate intestinal I/R injury (Cheng CH. et al., 2013). The formation of the mPTP and changes in mitochondrial membrane potential are key determinants of cell fate in I/R injury (Halestrap et al., 2002). Therefore, inhibition of mPTP formation is critical. In addition, apoptosis is an important mechanism of I/R-induced intestinal cell death. IPO reduces intestinal injury in rat models by inhibiting apoptosis of intestinal mucosal cells (Chu et al., 2015). This effect is mediated by the JAK/STAT pathway, which plays an important role in intestinal I/R injury (Wen et al., 2012). In a rat model of intestinal I/R, IPC has been found to induce upregulation of miR-21 through HIF-1α, inhibit apoptosis, and lead to downregulation of the apoptotic mediators PDCD4 and Fas-L, thereby mitigating intestinal I/R injury (Jia et al., 2017). Both miR-21 and HIF-1α are antiapoptotic miRNAs and factors that are upregulated during hypoxia, and further increases in the levels of these molecules during intermittent hypoxia may be responsible for the protective mechanism.

Remote ischemic preconditioning (RIPC) involves transient, repeated nonlethal ischemic treatment of organs or limbs to protect the distal organs/limbs from I/R injury (Hausenloy and Yellon, 2008). In addition to neurogenic pathways and systemic anti-inflammatory responses, the release of humoral mediators stimulated by RIPC may play a key role in its effects (Hausenloy and Yellon, 2008; Pickard et al., 2015). Several researchers have shown that RIPC reduces cell death from I/R injury in different organs (Cheng et al., 2014; Hu et al., 2016; Xu et al., 2017) and increases the antioxidant capacity of the tissue to mediate organ protection (Zitta et al., 2015; Jin et al., 2016; Motomura et al., 2017). Several studies have shown the beneficial effects of RIPC on the intestine (Candilio et al., 2013). IIn a rat model, Dickson et al. showed that RIPC increased intestinal tolerance to hypoxia after I/R injury. Saeki et al. found that in a rat model of small intestine transplantation, three cycles of 15 min of RIPC reduced intestinal injury (Toldo et al., 2018), while a single cycle had no significant protective effect on the hindlimbs (Yue et al., 2012). The potential mediators of RIPC include HIF-1α, ADAMTS1, Cited2, and cytochrome C, but the interactions of these molecules require further confirmation (Hummitzsch et al., 2019).

### Energy Therapy

ATP deficiency during intestinal I/R is the main cause of intestinal mucosal injury due to intestinal cell death. Providing enough ATP can ameliorate the disorder of the internal environment and maintain cell membrane stability. Mueller et al. (Mueller et al., 2018) injected creatine supplements into the intestinal lumens of SD rats to provide ATP for the ischemic intestine and observed that intestinal I/R could alleviate intestinal mucosal injury. Hansen et al. (Hansen et al., 2016) found that elevating mitochondrial levels in ischemic tissue can reduce intestinal mucosal damage induced by I/R. It may be useful to inhale two standard atmospheric pressures of oxygen and arterial infusion fructose diphosphate (FDP) during ischemia. This therapy reduces intestinal tissue damage by improving cellular energy metabolism and maintaining the integrity of the intestinal mucosal structure and function.

### Anti-free Radical Therapy

In many animal experiments, the use of antioxidants can significantly reduce I/R-induced tissue injury, and antioxidant free radicals are important treatments for intestinal I/R injury. Akgür et al. (Akgür et al., 1996) demonstrated that allopurinol inhibits the production of oxygen free radicals and attenuates intestinal mucosal injury after I/R injury. Mitochondrial oxidative damage may induce the release of mtDNA, activate pattern recognition receptors, and damage the intestinal barrier. Therefore, mitochondrial antioxidants can effectively prevent damage to intestinal barrier function. Recent studies have shown that pretreatment with the mitochondrial-targeted antioxidant MitoQ protects mitochondria from oxidative damage, reduces ROS production and stabilizes mitochondrial transcription factor A (TFAM) and that MitoQ activates the Nrf2/ARE pathway. MitoQ also prevents oxidative stress from damaging mtDNA, ultimately enhancing the integrity of the intestinal barrier (Hu et al., 2018b). Commonly used antioxidants include ATP, superoxide dismutase, glutathione, melatonin, vitamin C, vitamin E, and pyruvic acid.

### Anti-leukocyte Adhesion Therapy

When intestinal I/R injury occurs, the body initiates an inflammatory response. White blood cells are activated and adhere to the inner walls of blood vessels with microcirculation disorders, further leading to tissue and organ damage (Gordeeva et al., 2017). Gordeeva et al. (Gordeeva et al., 2017) found that inhibiting leukocyte activation, hindering leukocyte adhesion molecule synthesis, and reducing leukocyte-endothelial adhesion can reduce intestinal I/R injury. At present, there are leukocyte chemotactic drugs, such as PAF and anti-CD11 monoclonal antibodies.

### Glucocorticoids

Glucocorticoids (GCs) have strong effects on inflammatory and immune processes. They are commonly used to treat autoimmune and chronic inflammatory diseases (Cain and Cidlowski, 2017; Ronchetti et al., 2020). GCs are immunomodulatory, and acute exposure to GCs activates the immune system, whereas long-term use of GCs may lead to immunosuppression (Dhabhar, 2002). GCs exert anti-inflammatory effects by inhibiting proinflammatory cytokines and transcription factors and inhibiting anti-inflammatory gene activation (Ronchetti et al., 2015). Other studies have reported proinflammatory effects of GCs, such as induction of the expression of the NLRP3 inflammasome (Ronchetti et al., 2018). A large number of glucocorticoid receptor transcriptional effects contribute to the anti-inflammatory and immunomodulatory effects of GCs (Cain and Cidlowski, 2017). In the early stages of inflammation, circulating GCs can modulate cytokines involved in the immune response (Ji et al., 2016; Ronchetti et al., 2017). Therefore, GCs are widely used in the treatment of inflammatory diseases.

Synthetic GCs, such as prednisolone, betamethasone, and dexamethasone, are now commonly used clinically (Ronchetti et al., 2018). However, long-term GC use causes a wide range of adverse reactions (Ayroldi et al., 2014; Cain and Cidlowski, 2017), and rational use of GCs requires a deeper understanding of GC interactions in various systems. In addition, alternative molecules with the same efficacy as GCs with fewer side effects, such as glucocorticoid-induced leucine zipper (GILZ), have been shown to regulate immune cell activation and promote anti-inflammatory phenotypes (Berrebi et al., 2003; Ronchetti et al., 2020).

## Effects of Inflammation on Drug Transporters

Pathology may directly affect the functions of drug transporters (Drozdzik et al., 2020). Drug transporters, which are expressed in various cells, affect drug absorption, distribution, metabolism, and excretion (Müller et al., 2017). Drug transporter expression in the intestine and liver is critical in influencing oral pharmacokinetics and is directly related to drug efficacy and safety (Giacomini et al., 2010). The activities of drug transporters and drug-metabolizing enzymes are different in various pathological states. Here, we focus on the effects of inflammation on drug transporters.

Studies have shown that inflammation is an important factor altering drug action (Ling and Jamali, 2005; Clements and Jamali, 2007). Inflammation is associated with alterations in the gene expression of drug-metabolizing enzymes (Aitken et al., 2006), drug transporters, receptors (Hanafy et al., 2008), and plasma proteins. Hanafy et al. explored the overall effects of inflammation on target receptors using animal models of chronic inflammation. They found that inflammation led to significant decreases in the expression of the genes oct1, oatp4a1, and mrp1 in the liver; the genes oatp2b1, mrp6, and bsep in the kidneys; and the genes oct1, mdr1a, and mrp3 in the intestine. However, inflammation led to significantly elevated expression of the genes mdr1a and oatp4a1 in the heart (Hanafy et al., 2012). These molecular targets and transporters are associated with the pharmacodynamics and pharmacokinetics of some drugs. Therefore, inflammation alters the gene expression of drug transporters, which affects the expression and activity of drugs *in vivo*. However, little is known about the interactions between drug transporters and drug-metabolizing enzymes. The exact mechanism of the effect of inflammation on drug action remains to be investigated. In addition, studies on drug bioavailability in pathological states lack clinical validation.

## Effect of Increased Permeability of the Intestinal Barrier on Drug Absorption

The intestinal absorption of drugs involves multivariate processes, so predicting drug permeability in the intestine is difficult. Related articles have summarized the physicochemical parameters for estimation of intestinal permeability *in vivo* and *in vitro* models (Estudante et al., 2013). The limitations are that these methods do not consider the influences of physiological factors such as the gastric emptying rate and gastrointestinal transit rate. However, these methods can still be used as screening tools to assess gastrointestinal permeability.

Dixit et al. used hydrogen peroxide to induce oxidative stress to form a model of increased intestinal permeability. They further investigated how increased intestinal permeability affected drug absorption. They found that increased intestinal permeability elevated the apparent permeability of atenolol but had no effect on the apparent permeability of metoprolol (Dixit et al., 2012). Studies have demonstrated that atenolol is transported via the paracellular pathway (Brouwers et al., 2010), and that metoprolol is transported via the transcellular pathway (Hilgendorf et al., 2000). Therefore, the effect of increased intestinal permeability on the apparent permeability of drugs may be related to drug transport pathways, which needs to be supported by more research.

## Discussion

Intestinal I/R injury is an important problem in ischemic bowel disease. Mucosal damage and intestinal barrier disruption caused by I/R injury usually result in a systemic inflammatory response (Huang et al., 2011) and multiple organ dysfunction (Deitch et al., 2006). The intestinal tract is rich in mitochondria, which are among the most sensitive organelles to I/R injury. The pathogenesis of intestinal barrier dysfunction includes mitochondrial damage, inflammation, calcium overload, and free radical damage. Mitochondria and intestinal mucosal barrier function have a strong correlation. As an important DAMP, mtDNA has relatively low methylation and high sensitivity to oxidative damage. When released, mtDNA activates the TLR-9, NLRP3 inflammasome, cGAS-STING, and other signaling pathways. This induces inflammatory activation and the type I interferon response, which then damages the intestinal mucous membrane barrier.

In addition, mitochondrial oxidative stress produces many ROS and RNS. ATP synthesis is reduced, and the mitochondrial membrane potential changes, which is involved in intestinal apoptosis and necrosis and results in intestinal mucosal damage followed by intestinal mucosal barrier dysfunction. Damaged mitochondria can be cleared by mitophagy, preventing ROS accumulation and inflammation. In addition, mitochondrial antioxidant treatment is a feasible way to protect the function of the intestinal mucosal barrier. The intestinal mucosal barrier can also be protected by reducing intestinal I/R injury. Inflammation can alter the gene expression of drug transporters, thereby affecting drug absorption and activity *in vivo*. It is important to study the effects of inflammation on drug transporters. The effects of increased intestinal barrier permeability on various drugs may be closely related to drug transport pathways. More in-depth studies are needed to illustrate the effects of both inflammation and intestinal permeability on drugs.

Many questions related to intestinal I/R injury remain to be solved. For example, the mechanism of direct release of mtDNA through mitochondrial pores is not clear, and the methods for the prevention and treatment of intestinal I/R are not routinely used clinically. Looking to the future, the specific molecular mechanism between mitochondria and intestinal mucosal barrier dysfunction needs further study. Mitochondrial damage and mitochondrial release play important roles in the process of I/R injury. Further research to clarify the effects of mitochondrial DNA release on a variety of pathological processes may help with targeted organ protection.

## Abbreviations

DAMP, Damage-associated molecular pattern; MtDNA, Mitochondrial DNA; I/R, Ischemia/reperfusion; TLR, toll like receptor; NLRP, NOD like receptor; cGAS, Cyclic AMP-GMP synthase; STING, stimulator of interferon gene; mPTP, mitochondrial permeability transition pore; BAX, Bcl-2-associated X protein; BAK, Bcl-2 homologous antagonist/killer; SIRS, systemic inflammatory response syndrome; MODS, multiple organ dysfunction syndrome; MOMP, mitochondrial outer membrane permeabilization; MIMP, mitochondrial inner membrane permeabilization; PRR, pattern recognition receptor; MyD88, Myeloid differentiation primary response protein; ROS, reactive oxygen species; ATP, adenosine triphosphate; cGAMP, Cyclic guanosine monophosphate–adenosine monophosphate; TBK1, TANK-binding kinase 1; IRF3, Interferon Regulating Factor 3; IL, interleukin; NF-kB, nuclear factor-kB; IPC, ischemic preconditioning; IPO, ischemic postconditioning; RIPC, remote ischemic preconditioning; GCs, Glucocorticoids.
